# Mutational Signatures in Gastric Cancer and Their Clinical Implications

**DOI:** 10.3390/cancers15153788

**Published:** 2023-07-26

**Authors:** Pia Pužar Dominkuš, Petra Hudler

**Affiliations:** 1 Pharmacogenetics Laboratory, Institute of Biochemistry and Molecular Genetics, Faculty of Medicine, University of Ljubljana, Vrazov trg 2, 1000 Ljubljana, Slovenia; pia.puzar-dominkus@mf.uni-lj.si; 2 Medical Centre for Molecular Biology, Institute of Biochemistry and Molecular Genetics, Faculty of Medicine, University of Ljubljana, Vrazov trg 2, 1000 Ljubljana, Slovenia

**Keywords:** chromosomal instability, gastric cancer, genetic variability, gene expression, immune checkpoint inhibitors, microsatellite instability, mutational signatures

## Abstract

**Simple Summary:**

There is a lack of molecular biomarkers that would allow better characterisation and categorisation of gastric tumours. Distinct mutational patterns have been observed at both the whole genome and exome levels and have been referred to as mutational signatures. Some of these characteristic mutational patterns have been associated with defects in DNA repair mechanisms or linked to exogenous mutagens. The mutational signatures found in gastric tumours could be used as prognostic biomarkers and could provide new information about the drivers of gastric carcinogenesis, which might be useful for the improvement in disease treatment options. This review summarises mutational signatures found in gastric cancer and their clinical potential.

**Abstract:**

Gastric cancer is characterised by high inter- and intratumour heterogeneity. The majority of patients are older than 65 years and the global burden of this disease is increasing due to the aging of the population. The disease is usually diagnosed at advanced stages, which is a consequence of nonspecific symptoms. Few improvements have been made at the level of noninvasive molecular diagnosis of sporadic gastric cancer, and therefore the mortality rate remains high. A new field of mutational signatures has emerged in the past decade with advances in the genome sequencing technology. These distinct mutational patterns in the genome, caused by exogenous and endogenous mutational processes, can be associated with tumour aetiology and disease progression, and could provide novel perception on the treatment possibilities. This review assesses the mutational signatures found in gastric cancer and summarises their potential for use in clinical setting as diagnostic or prognostic biomarkers. Associated treatment options and biomarkers already implemented in clinical use are discussed, together with those that are still being explored or are in clinical studies.

## 1. Introduction

Gastric cancer was rated as the fifth most common cancer type and the fourth most common cause of cancer deaths worldwide [1]. The full extent of the global burden of this disease and public health impact is hard to estimate, particularly in the light of emerging demographic shifts in age distribution toward older populations in developed countries [2,3]. In these countries, where the gastric cancer cases are predominantly sporadic, the majority of patients are diagnosed between ages 65 and 89 [4,5]. Global changes in the demographic structure of the populations in these countries, particularly the fast growth in the population segment that comprises individuals over 65 years, is indicating that the incidence of this devastating disease could rise by 62% by the 2040 [6]. This will have great impact on public health services and costs as well as on the quality of life of elderly affected individuals, considering that the clinical practices for gastric cancer treatment can be challenging in older individuals, who often have other comorbidities. The development of gastric tumours is mostly characterised by common, nonspecific symptoms and the progress of the disease is often overlooked due to the late presentation of clinically relevant symptoms [7]. The late diagnosis contributes to the obstacles in cancer management of this life-limiting disease, since the tumours may have already spread to the nearby nodes and peritoneum, and to nearby or distant organs and are often treatment-resistant [8,9].

Despite the steady decline in gastric cancer incidence from the early 1900s, which was attributed to the socioeconomic development and improvements in food preservation practices, prompting the decrease in smoked and salted food consumption, it has remained the leading cause of death up until 1980 [5,6]. Recent analyses indicated that significant progress in cancer control, such as increased survival, decreased mortality, and incidence, has been evident for some poor prognosis cancers, including stomach cancer [10]. The favourable trends were most substantial for patients who were younger than 75 years at the diagnosis. This has been attributed to the improvements in diagnosis, treatment, and *Helicobacter pylori* eradication. 

However, no major improvements have been made at the level of noninvasive molecular diagnosis of sporadic gastric cancer. Genetic diagnostic approaches are available for only approximately 10% of gastric cancers, most of which are being used to identify known inherited cancer predisposition syndromes. Despite remarkable advances in the post-genome era and numerous studies researching different biological aspects of gastric cancer, only a few molecular predictive biomarkers have been implemented in clinical practice. The first was tyrosine kinase receptor HER2, which is overexpressed in up to 25% of gastric cancers [8]. ToGa study, performed in 2010, invariably proved that patients, whose tumours overexpressed HER2, benefited from targeted therapy with HER2 inhibitors, such as trastuzumab [11]. Another established biomarker is programmed death ligand 1, PD-L1, which is often overexpressed in tumours that also display microsatellite instability (MSI) [12]. Several clinical trials, for example, KEYNOTE-012 and KEYNOTE-059, proved the efficacy of immune checkpoint inhibitors, targeting PD-L1, such as pembrolizumab, for the treatment of MSI-high gastric tumours overexpressing PD-L1 [13,14]. It should be noted that recent research showed that approximately half of MSI-high/high tumour mutational burden (TMB) patients are resistant to pembrolizumab therapy, indicating that more complex diagnostic tools should be developed in order to select MSI-high patients, who would respond to pembrolizumab monotherapy, or select MSI-high patients who would be susceptible to pembrolizumab therapy in combination with additional therapeutic approaches [15,16]. These diagnostic methods would probably encompass genomic-paired tumour tissue analyses combined with T-cell population analyses in peripheral blood. Based on the results of the REGARD trial, ramucirumab, a VEGFR2 antagonist, was approved for the treatment of advanced gastric cancer, not responding to the first line of treatment [17]. However, the immunohistochemical evaluation of VEGFR2 expression in tumour tissues has little or no predictive value, therefore it is currently not recommended as a diagnostic tool [18]. The same applies to the EGFR (epidermal growth factor receptor) inhibitor, which was overexpressed in 27–55% of tumours and was associated with lower patient survival [19,20]. A monoclonal antibody against the EGFR is being studied as a therapy option, although it has not shown the desired efficacy so far. Studies that are examining inhibitors of the protein claudin 18.2, which is highly expressed in the primary tumour and lymph node metastases, appear more promising at present [21].

With the exception of established, relatively nonspecific biomarkers, such as carcinoembryonic antigen (CEA), cancer antigen 19-9 (CA19-9), and cancer antigen 72-4 (CA72-4), there are currently no noninvasive diagnostic, prognostic, or predictive biomarkers that can be detected in the blood of patients with gastric cancer in order to diagnose and stratify patients to receive particular therapies, or for modelling the disease progression. This is the consequence of the fact that sporadic gastric cancers are immensely complex and heterogeneous in terms of the underlying molecular pathogenic changes. One of the most prominent molecular features of sporadic gastric cancer is high inter- and intratumour genetic heterogeneity. The Cancer Genome Atlas (TCGA) study identified four main molecular subtypes of gastric cancer that are characterised by distinct molecular features [22]. Some of these protein biomarkers are currently being explored as targets for drug discovery. Another promising avenue stemming from this study is that molecular characteristics separating these subtypes could serve as the basis for the development of new diagnostic tools for early detection of the disease. In another large study, the Asian Cancer Research Group (ACRG) also identified four subtypes of gastric cancer based on gene expression data, which were characterised by specific genomic mutational signatures, survival outcome, clinical phenotype, and recurrence after surgery [23]. 

New approaches and biomarkers are urgently needed for early diagnosis, effective patient stratification, and targeted therapeutic strategies. Advancements in the fields of sequencing technologies, bioinformatics, and single cell analysis generate large amounts of data and bring new possibilities for better understanding and classifying malignant disease. In recent years, an exciting new field emerged in the area of genomics that describes the patterns of mutation in human (tumour) genome–mutational signatures. In this review, we aim to inspect the current information about mutational signatures in gastric cancer (at the whole genome/exome level) and discuss how this knowledge can be relevant for its translation and implementation into clinical practice.

## 2. Mutational Signatures

In nonhereditary cancers, the accumulation of somatic mutations in cells leads to clonal expansion and malignant transformation. Mutations occur in the genome due to exogenous and endogenous mutagens in the presence of normally or abnormally functioning DNA maintenance machinery. The ones that occur in critical genes, which maintain cell integrity and result in cell growth advantages, are known as “driver” mutations. At the same time, many other mutations accumulate in the genome regions with no result in functional or phenotypic change, so-called “passenger” mutations. Each exo- and endogenous mutational process leaves a distinct mutational pattern of both driver and passenger mutations on the genome—termed “mutational signature” (in some publications the term mutational fingerprint is used). In the past, specific mutational patterns of environmental agents were already observed in single genes *TP53* and *BRAF* [24,25,26]. On the whole genome level, however, this was first comprehensively described by Nik-Zainal et al. and Alexandrov et al. [27,28]. Alexandrov et al. analysed mutations from 7042 primary cancers (paired normal–tumour samples) and looked for distinct patterns in single base substitutions (SBS) [29]. Twenty-one such patterns were recognised and named mutational signatures. The analysis of sequencing data from a large number of tumour samples was made possible with a computational framework that used nonnegative matrix factorisation (NMF) for the recognition of multiple base substitution patterns [28]. In the following years, more mutational signatures were identified by analysing large series of whole-genome (WGS) and whole-exome (WES) sequencing data [30,31,32,33,34,35]. There are currently a total of 110 mutational signatures (excluding artefacts) catalogued in the COSMIC database (https://cancer.sanger.ac.uk/cosmic/signatures/, v3.3—accessed on 23 June, 2022) [36]. 

In the category of base substitutions, single base substitution (SBS) and doublet base substitution (DBS) have thus far been studied. For SBS, a signature was characterised by a specific base change (C·G→A·T, C·G→G·C, C·G→T·A, T·A→A·T, T·A→C·G, and T·A→G·C) and information concerning its 5′ and 3′ adjacent bases [31]. “There are six classes of base substitutions and 16 possible trinucleotide sequence contexts, which results in 96 possible combinations (e.g., A[C > T]A, A[C > T]T, etc.). It the case of SBS +2, two neighbouring bases 5′ and 3′ to the mutated base are considered, generating 1536 classes”. Regarding the DBSs, “there are 16 possible source doublet bases (4 × 4) [31]. Of these, AT, TA, CG, and GC are their own reverse complement. The remaining 12 can be represented as 6 possible strand-agnostic doublets. Thus, there are 4 + 6 = 10 source doublet bases. Because they are their own reverse complements, AT, TA, CG, and GC can each be substituted by only 6 doublets. For the remaining doublets, there are 9 possible DBS mutation types (3 × 3). Therefore, in total there are 4 × 6 + 6 × 9 = 78 strand-agnostic DBS mutation types”. Other categories of mutational signatures have been observed, such as small insertions and deletions (indels, ID), structural variants, and clustered mutational signatures [30,31,37,38]. In the COSMIC database, small insertions and deletions are defined “as the incorporation or loss of small fragments of DNA (usually between 1 and 50 base pairs) in a specific genomic location. A compilation of 83 different types considering size, nucleotides affected and presence on repetitive and/or microhomology regions was used to extract mutational signatures” [31]. Copy number (CN) variation signatures are defined “by using the 48-channel copy number classification scheme. The scheme incorporates loss-of-heterozygosity status, total copy number state, and segment length to categorise segments from allele-specific copy number profiles (as major copy number and minor copy number, respectively, i.e., nonphased profiles)” [35]. Profiles of and information on 60 SBS, 11 DBS, 18 ID, and 21 CN signatures are available from the COSMIC database (https://cancer.sanger.ac.uk/cosmic/signatures/ v3.3—accessed on 23 June 2022) [36]. 

Mutational signatures are correlated to specific endo- or exogenous mutagenic processes, such as spontaneous deamination of C > T due to aging, overactivity of APOBEC cytidine deaminase enzymes, defects in DNA repair machinery, tobacco smoking, and so on. They are more precisely discussed later in this review. Some mutational signatures are rare and specific, found only in distinct cancer types, for example, mutational signature resulting from UV light exposure found in skin melanoma and to a small extent in head and neck squamous cell carcinoma, whereas others are more common and can be detected in most cancer types, such as mutational signatures resulting from reactive oxygen species (ROS), APOBEC overactivity, and defective mismatch repair (MMR) [31]. The underlying mechanisms remain unknown for many mutational signatures and further in vitro experiments on cell lines, exposed to mutagenic processes in defined environments, are necessary to decode the causal mutagenic factors. One such comprehensive study used WGS to analyse mutational signatures of human-induced pluripotent stem cells, which were exposed to 79 known or suspected environmental carcinogens [32]. Some of these imprints were similar to cancer-derived signatures linked to specific mechanisms and carcinogens, such as compromised DNA maintenance machinery, tobacco smoking, and UV light exposure. Other compounds generated signatures with weaker similarities to cancer-derived signatures. However, the authors stressed that mutational outcomes probably depend on the tissue type and cellular genetic background [32]. Therefore, additional studies on different cell lines are desirable in order to provide more comprehensive data, which could be used to compile detailed carcinogen-related mutational profiles. Decoding and understanding the cancer mutational signatures would provide an additional level of insight into tumour aetiology and has many implications for clinical use in cancer prevention and treatment. 

## 3. Mutational Signatures in Gastric Cancer

**Figure 1:**
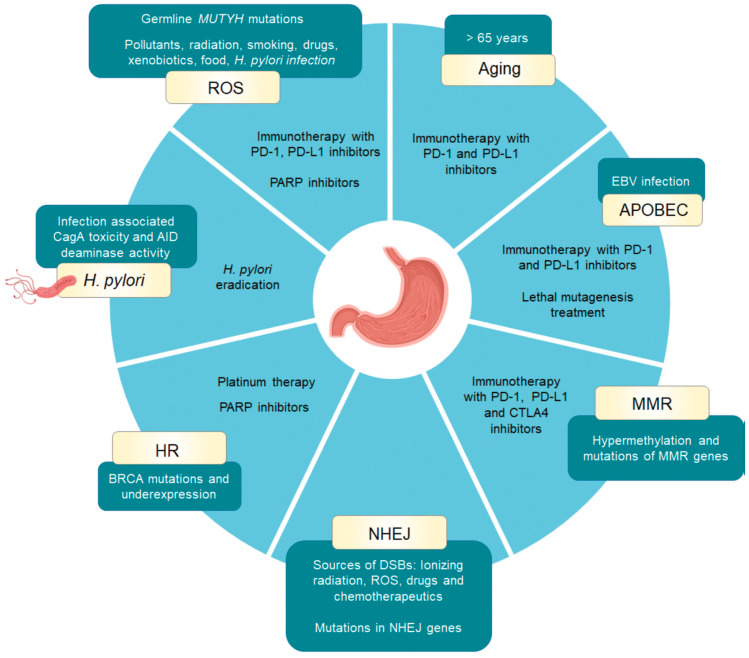
Risk factors and current treatment possibilities associated with gastric cancer mutational signatures. Mutational signatures found in gastric cancer are represented in yellow boxes. Corresponding risk factors are represented in green boxes and current treatment possibilities are listed in the circle diagram. For NHEJ inhibition, no treatment options are currently in clinical use. HR, homologous recombination; MMR, mismatch repair: NHEJ, nonhomologous end joining; ROS, reactive oxygen species. Illustrations created with BioRender.com.

Gastric cancer falls into the category of cancer types with a complex repertoire of mutational processes. In this section, we included WES and WGS mutational signatures found in 75 samples from gastric adenocarcinoma tumours by Alexandrov et al., which are also described in the COSMIC database [31,36]. We discuss their possible implications in gastric cancer prevention, diagnosis, and treatment (Table 1 and Figure 1).

### 3.1. Aging

SBS1 mutational signature is ubiquitous across different cancer types, including gastric cancer and normal cells [31]. It has been associated with a continuous mutational process that occurs throughout life in normal tissues at constant rates in all individuals. Its feature is spontaneous hydrolytic deamination of 5-methylcytosine related to aging [39,40]. The SBS1 profile is distinguished by C > T mutations at NpCpG trinucleotides (mutated base underlined; N any base), and specifically within CpG motifs, where C is methylated [29,86]. Spontaneous or cytidine deaminase mediated deamination of cytosine and methylated cytosine result in U:G and T:G mismatches, respectively. Studies have shown that methylated cytosine is more sensitive to deamination than intact cytosine [87]. Uracil is readily repaired in cells with functional uracyl-DNA glycosylase and base excision repair, whereas T:G base pairing is recognised by thymidine-DNA glycosylase. Evidence showed that T:G mismatches, particularly in the context of CpG islands, are often associated with mutational hotspots in certain genes, such as *TP53* [88]. Interestingly, when comparing signature SBS1 mutation rates between different tissue types, they were the highest in stomach (23.7 mutations per gigabase per year) followed by colorectum (23.4 mutations per gigabase per year), whereas the rates were the lowest in breast tissues (3.1–3.9 mutations per gigabase per year) [40]. This is attributed to high mitotic rates of gastric and colon epithelia, where cells completely turnover every 2–7 days under physiological conditions [89]. Although this is a universal signature that may be correlated with the age of the patient and perhaps to some extent with the mitotic rate of tissue of origin, it could still be clinically relevant in combination with other markers of senescence. It could perhaps be included in the senescence scoring models, with potential prognostic values. Zhou et al. constructed a senescence scoring system based on six genes (*ADH1B*, *IL1A*, *SERPINE1*, *SPARC*, *EZH2*, and *TNFAIP2*) and showed that patients with high senescence score (senoscore) had longer overall survival (19.6 months vs. 56.2 months; *p *<  0.0001) [43]. Furthermore, when patients were stratified according to TNM (tumour node metastasis) clinical stages, the senescore successfully separated patients with distinct clinical prognosis at the same disease stage. High senoscore was also related to the MSI-high status, Epstein–Barr virus (EBV) infection, and higher TMB, suggesting that these patients would benefit from immune checkpoint inhibitors, such as PD-1 and PD-L1 [16,41,42]. It has been observed that patients with high senoscore may experience stronger adverse effects after chemotherapy and cancer relapse [90]. Therefore, research results suggested that patients with low senoscore would be better candidates for chemotherapy. 

### 3.2. APOBEC Activity

SBS2 and SBS13 mutational signatures are found in more than half of all cancer types including gastric cancer and are ranked second in cancer mutagenesis, with aging being the first one [29]. They are associated with the activity of the activation-induced cytidine deaminase/apolipoprotein B editing complex (AID/APOBEC) enzyme family [29]. Signature DBS11 is also found in samples with a large number of SBS2 and SBS13 mutations, although it has been correlated with ROS based on experiments in bacterial models [44]. The AID/APOBEC family comprises eleven members with distinct functions. These enzymes deaminate cytidine to uridine in the DNA and/or RNA, and are implicated in adaptive (antibody gene diversification) and innate immunity (virus restriction) as well as in retrotransposon restriction. Their characteristics and functions have been reviewed in detail by Viera et al. and Conticello et al. [91,92]. A side effect of APOBEC overexpression is off-target genomic mutations, which accumulate in the host DNA, affect the DNA integrity, and lead to neoplastic transformation. APOBEC mutational signature is characterised by C > T transition mutations (SBS2) in DNA motifs TpCpW and C > G transversions in SBS13 in TCW DNA motifs (mutated base underlined; W = A or T), and other mutational outcomes that occur due to DNA repair intermediates such as abasic sites and DNA breaks [93]. The substitutions occur during replication of uracils formed by APOBEC cytidine deamination and by error-prone polymerases following uracil excision and generation of abasic sites by uracil–DNA glycosylase [94,95].

In viral infections, APOBEC mutates viral ssDNA/ssRNA in order to hamper virus replication and function [91]. Viral infections are associated with the development of a number of cancers. Hepatitis B virus (HBV) is one of the leading causes of liver cancer; human papilloma virus (HPV) causes anal, cervical, penile, oropharyngeal, vaginal, and vulvar cancer; human T-lymphotropic virus type 1 (HTLV-1) is associated with adult T-cell leukemia/lymphoma; hepatitis C virus (HCV) with liver cancer and non-Hodgkin’s lymphoma; and EBV with the risk of Burkitt lymphoma, some types of Hodgkin’s and non-Hodgkin’s lymphoma, and, importantly, gastric cancer. According to the TCGA study on 295 primary gastric adenocarcinoma samples, around 8.8% were characterised as EBV positive (EBV^+^) with distinct molecular characteristics—mutations in *PIK3CA* and *ARDN1A*, DNA hypermethylation, overexpression of PD-L1/2, amplification of *ERBB2* and *JAK2*, low rate of *TP53* mutations, and intestinal subtype [22]. In addition, strong IL-12 signalling indicated that EBV^+^ tumours were infiltrated with immune cells. In the ACRG study, using different approaches to molecularly stratify gastric cancer subtypes, the researchers observed that EBV infection occurred more frequently in the MSS/TP53^+^ group (*n* = 12/18 of EBV^+^) [23]. Overall, in their cohort, 6.5% samples were characterised as EBV^+^. They also observed frequent *PIK3CA* and *ARDN1A* mutations and a distinct cytokine signature, indicative of increased infiltration of immune cells in EBV^+^ tumours compared to microsatellite stable (MSS) tumours. The predominant histology of tumours in this group was also intestinal-type carcinoma. They did not observe alterations in *TP53*, which is partly consistent with the finding from the TCGA study, where the majority of EBV^+^ tumours had intact *TP53*. Interestingly, in the ACRG study, *MDM2* was amplified in MSS/TP53^+^ cancers, whereas in the TCGA study it was not.

Bobrovnitchaia et al. analysed 240 gastric cancer samples from the TCGA cohort and 112 samples from a different Brazilian validation cohort and showed that the expression of *APOBEC* genes was significantly higher in EBV^+^ tumours in comparison to EBV^−^ tumour samples [45]. With the exception of *APOBEC3A*, all other members of the *APOBEC3*s were upregulated, with *APOBEC3C* being the most abundantly expressed. The authors also showed that the *APOBEC* characteristic TpCpW mutation pattern was significantly enriched in the EBV^+^ group and positively correlated with *APOBEC3* expression, which further correlated with tumour purity, suggesting that APOBEC activity derives from tumour cells. APOBEC mutational load correlated with highly expressed genes, which were, as expected, highly mutated. This is in accordance with the fact that the preferential editing substrate of APOBEC3s is ssDNA, which is available at transcriptionally active sites. The authors also observed enrichment in TpCpW mutations in the late-stage EBV^+^ tumours, which also carried a higher proportion of mutated oncogenes in comparison to the EBV^−^ samples. Furthermore, in 40% of EBV^+^ patients with somatic mutations in PIK3CA, mutations were present in the TpCpW motif, while this was not observed in EBV^−^ patients. 

Higher expression patterns in mRNA and protein levels of APOBEC3B were also found in gastric cancer tumour samples compared to paired normal tissue samples in a cohort of 236 patients [96]. *APOBEC3B* mRNA and protein levels were higher in tumour samples from Grade III stage in comparison to Grade I or Grade II stage and correlated to poor prognosis. High *APOBEC3B* expression was also associated with gender (female), tumour size (>5.0 cm), histological grade (G3), and TNM staging (lower expression in TNM I). *APOBEC3B* downregulation with shRNA resulted in enhanced cytotoxicity of PDCD2 (programmed cell death protein 2) in gastric cancer cell line MKN28, probably due to the lower mutational load and noninterfered transcription of this tumour suppressor gene. *APOBEC3B* expression levels were associated with APOBEC mutational signatures, whereas *APOBEC3C* expression levels were associated with decreased APOBEC mutational signatures in gastric cancer [46].

Interestingly, analysis of cancer cell lines by sequencing single cells showed that at least 75% of investigated cancer cell lines (including gastric cancer) that previously encountered APOBEC mutagenesis persistently continued to generate SBS2 and SBS13 mutational signatures [47]. The authors suggested that APOBEC-associated mutagenesis in vivo appears to be episodic. Additionally, the procurement of APOBEC-associated mutational signatures continued in cell culture despite the absence of proposed initiators of APOBEC activity, immune system and exogenous viral infections [91,97]. Indeed, the presence of a virus is not necessarily required for initiating APOBEC mutagenesis [47]. This may also occur due to the retrotransposition activities. There was significant correlation between the rates of the in vitro-acquired retrotransposition aberrations and burdens of SBS13 and, interestingly, SBS18 (associated with ROS) shown in cancer cell lines. However, the correlation was not significant when 2353 primary cancers originating from different tissues were investigated. L1s are autonomous mobile elements, amounting to 17% of the human genome, and retrotranspose via an RNA intermediate through a “copy and paste” mechanism [98]. Somatic mobilisation of retroelements can induce and accelerate insertional mutagenesis and genetic instability. Next-generation L1-resequencing (L1-seq) on paired tissue samples from seven patients with primary gastric cancer showed that somatic retrotransposition was present early in cancer development in gastrointestinal epithelial cells [52]. L1-seq, APOBEC-mutational signature, and APOBEC-expression status in stomach tissues in combination with circulating biomarkers could therefore be valuable as biomarkers for early detection of gastric cancer.

Tumours characterised by APOBEC overactivity might be candidates for treatment by lethal mutagenesis [48]. Drugs, such as nucleoside analogues that increase the mutation load in tumour cells to toxic levels, are already commonly used in addition to platinum-based chemotherapy to destroy tumour cells. On the other hand, inhibition of APOBEC enzymes may prevent cancer evolution. APOBEC3B specific inhibitors are promising, as this enzyme is nonessential in humans, whereas other members of the family are crucial for adaptive (AID) and innate (other APOBEC members) immune response [49]. Drug candidates for such “therapy by hypomutation” are being pursued. In recent years, numerous studies of APOBEC3B crystal structures have been published and provide a better understanding of APOBEC3B active site dynamics, setting the stage for design of selective small molecule inhibitors [99,100,101]. In combination with other therapies, these may in the future be effective in preventing tumour recurrence and drug resistance.

Furthermore, a study by Wang and Jia et al. suggested that APOBEC3B and APOBEC-mutational signature might be a novel marker for predicting immunotherapy response [50]. They found a correlation between *APOBEC3B* expression and immune gene expression and known immunotherapy response biomarkers in patients with nonsmall cell lung cancer. APOBEC mutational signature was specifically enriched in patients with lasting clinical benefit after immunotherapy, suggesting that patients with APOBEC signatures might be candidates for checkpoint blockade immunotherapy with PD-1 and PD-L1 (discussed in detail in Section 3.3.2). Similarly, Boichard and Pham et al. demonstrated that APOBEC-related mutagenesis could be correlated with immunotherapy response in patients with various cancer types (gastric cancer excluded) [51].

All of the factors responsible for the expression of the AID/APOBEC enzyme family are currently unknown. Nevertheless, altogether these data suggest that it might be worth analysing the presence of APOBEC mutational signature and the expression levels of APOBEC enzymes in gastric cancer biopsies as they can be a source of ongoing mutagenesis at transcriptionally active DNA sites or retrotransposable elements, which provide a secondary driving force for subclonal expansions and intratumour heterogeneity propagation. This may manifest clinically as recurrence, metastasis, and drug resistance and could therefore have important prognostic and therapeutic implications. In a cohort analysed by Alexandrov et al., APOBEC signatures were present in <50% of all gastric cancer samples, indicating that this feature was not common for all gastric adenocarcinoma tumours and could therefore be useful for patient stratification in combination with other markers, such as PD-L1 positivity, TMB, and EBV infection status [31].

### 3.3. DNA Integrity Machinery

Compromised DNA replication and repair mechanisms play a central role in cancer development. Deficient DNA integrity machinery leaves distinct imprints in the genome. Therefore, it is no surprise that several mutational signatures were associated with aberrations of specific DNA repair mechanism or a specific DNA repair gene [31]. Inhibitors of compromised DNA repair pathways are promising drugs for cancer treatment and could be used as monotherapy or in combination with first-line chemotherapeutics to increase tumour mutational burden. However, it is difficult to select the patients who would benefit from a particular combination therapy due to the lack of specific biomarkers. Mutational signatures could be a promising tool for filling the gap in biomarker selection for better patient stratification in gastric cancer.

#### 3.3.1. Homologous Recombination DNA Repair

SBS3 was associated with germline or somatic mutations in *BRCA1* and *BRCA2,* and *BRCA1* promoter methylation [27,29]. BRCA1/2 tumour suppressors play an important role in the response to DNA damage, particularly DNA double-strand breaks (DSBs), which are usually repaired by error-free homologous recombination repair (HRR) [102]. Additionally, they maintain genome integrity by chromatin remodelling, and transcriptional and cell cycle regulation [103]. Defects in BRCA1/2 lead to activation of alternative error-prone DNA repair mechanism by nonhomologous end joining (NHEJ) [104]. Consequently, cells have higher mutational burden, which in time leads to neoplastic transformation. *BRCA1/2*-associated mutational signature is commonly accompanied by small deletions with overlapping microhomology at their boundaries (specified as ID6) and large numbers of rearrangements, such as tandem duplications (short tandem duplications (1–10 kb)) and (longer tandem duplications (>100 kb)) as well as indels (deletions (1–10 kb)) [27,30,105]. SBS3 is very common in breast, ovarian, and pancreatic cancer with mutations in *BRCA1/2* genes; however, it is also present to a smaller extent in other cancer types with no mutations in *BRCA1/2* or other genes involved in double-strand break repair [31]. 

Alexandrov et al. analysed the data from 372 whole-exome and 100 whole genome sequences from gastric cancer patients and showed that SBS3 is present in 7.3% of the examined whole-exome and in 12.0% of the examined whole-genome gastric samples [53]. Samples with SBS3 had statistically significant elevation in large indels with overlapping microhomologies and structural rearrangements, and were enriched in the intestinal type by Lauren’s classification. Interestingly, although some gastric samples harboured *BRCA1* or *BRCA2* somatic mutations, they were not enriched with SBS3, suggesting that these mutations may actually derive from defective MMR.

The authors suggested that gastric cancer patients with SBS3 might benefit from platinum therapy or PARP inhibitor treatment since this approach was beneficial in breast, ovarian, prostate, primary peritoneal, and pancreatic cancers with defective DSB repair due to BRCA1/2 mutations, HR mutations, or high genomic instability score [53]. Platinum–DNA adducts are genotoxic and BRCA1/2-defective neoplastic cells undergo apoptosis as DNA damage accumulates and cannot be efficiently repaired. PARP inhibitors mediate selective cytotoxicity as they introduce even more DSBs in tumour cells with deficient HRR by inhibiting PARP1, responsible for single-strand break repair. When these remain unrepaired, DSBs are formed during DNA replication. It has been established that breast and ovarian cancers with defective BRCA1/2 benefit from treatment with a range of PARP inhibitors [54,55]. In addition, a study on WGS data from pancreatic cancer samples revealed that patients with SBS3 responded to platinum therapy [56]. Recently, this approach has also been proven successful for small cell lung cancer [57]. 

There are several currently ongoing preclinical and clinical trials for PARP inhibitors as monotherapy or combination therapy. A comprehensive review on PARP inhibitors in gastric cancer has been recently published [106]. Since gastric tumours are extremely heterogeneous, PARPis may have higher efficiency in gastric tumours with specific biomarkers, such as the use of ATM loss as a predictive biomarker of tumour response [58]. Additional studies with patient stratification based on BRCA1/2 status, ATM-status, defective HRR, and SBS3 presence may result in a different outcome. In addition to comprehensive selection of biomarkers for patient stratification, drug mechanisms and, particularly, its consequences and effects on the DNA, should be carefully considered in future research.

Several studies have already observed association between *BRCA1* expression and gastric cancer. Gastric cancer patients with BRCA1-negative status benefited from platinum-based adjuvant chemotherapy (*p* = 0.024) in comparison with patients with BRCA1-positive expression [59]. Negative or reduced expression of BRCA1 was more common in more advanced stages of the disease (*p* < 0.001) and was associated with perineural invasion (*p* = 0.032) [60]. Disease-free survival was significantly decreased with reduced BRCA1 expression (*p* = 0.027), suggesting that negative BRCA1 nuclear expression could be a predictive marker for the stratification of sporadic gastric cancer patients, who would be good candidates for the adjuvant chemotherapy. Adjuvant chemotherapy indeed enhanced disease-free survival and overall survival in stage III patients, who had only BRCA1-negative tumours (*p* < 0.001, *p* < 0.001, respectively), but not in patients with BRCA1-positive tumours (*p* = 0.236, *p* = 0.148, respectively). 

There is an ongoing debate about non-BRCA-mutant tumours, which exhibit BRCAness and HRR deficiency, and their sensitivity to PARP inhibitors [55]. Patient stratification based on the presence of SBS3 mutational signature and accompanying indels and rearrangements as a biomarker would perhaps be more effective and would provide additional information to BRCA1/2 mutational/expression status. Further large studies that would examine the benefit of platinum and PARP inhibitor treatments in gastric cancer patients with SBS3 in addition to other biomarkers are necessary to confirm this association. 

#### 3.3.2. DNA Mismatch Repair Deficiency

SBS6, SBS14, SBS15, SBS20, SBS21, SBS26, and SBS44 were strongly associated with deficient MMR and microsatellite instability [31]. The common characteristics of these mutational signatures are presented in Table 2. Interestingly, the majority of patients with gastric cancer were characterised by SBS15 (60/486) and SBS20 (56/486), followed by SBS44 (17/486), SBS26 (11/486), SBS6 (9/486), and SBS21 (5/486). SBS14, which was associated with *POLE* mutations in addition to MMR deficiency, was found in only one gastric cancer sample (1/486). All mutational signatures were strongly associated with ID1 and ID2 [36]. The SBS profiles and the additional data on the transcriptional and replicational strand symmetry could either be the consequence of sequencing artefacts or it could reflect chemotherapy or carcinogen exposure, or could also indicate the underlying biological mechanisms leading to specific mutation profile. Therefore, distinct mechanisms could contribute to the differences in base substitutions. In particular, SBS20 was conjoined with mutations in *POLD1* and was characterised mainly by C > A substitutions in CpCpT and CpCpC and less by C > T substitutions (discussed in more detail in the next section). This is in contrast with SBS15, which is distinguished predominantly by C > T substitutions in the GpCpN context. Next, indications that some substitutions are asymmetrically distributed between leading and lagging strand and between transcribed and untranscribed strand could lead future research to identify aberrations in cell processes, implicated in this specific mutator phenotype [31,36]. Approximately 22% of examined tumours in the TCGA study and ACRG study were classified as microsatellite unstable (MSI) tumours, displaying numerous mutations in receptor tyrosine kinase (RTK) and RAS signalling pathways (*EGFR*, *ERBB2*, *ERBB3*, *JAK2*/*PD-L1*/*2*, *FGFR2*, *MET*, *VEGFA*, *KRAS*/*NRAS*, *RASA1*) and in the PI(3)-kinase pathway (*PIK3CA* and *PIK3R1*) and frequent truncating mutations in *PIK3R1* and *PTEN* [22,23]. Therefore, activation of different cell processes, such as promoting cell division and/or transcription, could in combination with other accumulated aberrations result in the observed asymmetry of mutational signatures on the DNA strands. 

**Table 2:** Characteristics of MMR-associated mutational signatures.

SBS	Base Substitution Subtype ^1^	Associated ID	Transcriptional (T) and Replicational (R) Strand Asymmetry in Stomach Cancer
SBS6	C > T (ACA, ACG, CCG, GCN)	ID1, ID2	T:/ R:/
SBS14	C > A (ACT, CCT, GCT, TCT)	ID1, ID2	T:/ R:/
SBS15	C > A (CCA) C > T (ACG, GCN)	ID1, ID2	T:/ R:/
SBS20 ^2^	C > A (CCC, CCT) C > T (ACA, GCA, GCC)	ID1, ID2	T: no significance R: lagging, C > A
SBS21	T > C (GTN, TTA, TTC, TTT)	ID1, ID2	T: no significance R: lagging strand, T > C
SBS26	T > C (ATA, ATC, CTA, CTG, CTT, GTA, GTG, GTT, TTT)	ID1, ID2	T: untranscribed strand, T > C R: lagging strand, T > C
SBS44	C > A (CCT) C > T (ACA, GCN)	ID1, ID2	T: no significance R: lagging strand, C > A; leading strand, C > T and T > A

^1^ Only base substitutions with more than 5% of percentage of single base substitutions ^2^ Characterised by *POLD1* mutations.

##### MLH1 Promoter Hypermethylation and MSI Phenotype

The main mechanism contributing to MSI phenotype in sporadic gastric cancers is hypermethylation of *MLH1* promoter [22]. MSI phenotype in gastric cancer has favourable prognosis, particularly for women; however, analysis of several clinical trials (MAGIC, CLASSIC, ARTIST, and ITACA-S) indicated that high MSI status (MSI-high) was negatively associated with the efficacy of adjuvant or neoadjuvant chemotherapy in gastric cancer patients [69]. Interestingly, some of the MSI tumours also harboured frequent common alterations in major histocompatibility complex class I (*MHC I*) genes, suggesting that alterations in *MHC I* genes, which play a crucial role in antigen presentation, could contribute to tumour evasion from the immune response. It has been postulated that this subset of patients could benefit from immune-based therapies [22,107]. Several studies of different cancer types showed that high tumour mutational burden, MSI, and PD-1/PD-L1 immunohistochemical status in tumour tissues could identify patients who would respond to immune checkpoint inhibitors (ICIs). ICIs have emerged as a promising new treatment option for gastric cancer patients as well, improving the 12-month and 18-month overall survival (RR, 1.79 *p* = 0.013; 2.20 *p* = 0.011) in patients with advanced and metastatic stomach adenocarcinomas [62,63,64]. 

Buttura et al. used a different computational approach than Alexander et al. to obtain mutational signatures from data combined from several nonredundant cohorts of patients with gastric cancer [61]. They identified seven mutational signatures that partly corresponded to COSMIC mutational signatures. Signatures S2, S4, and S5 were associated with defective mismatch repair (dMMR) and/or MSI and were similar to SBS6 and SBS15, SBS20, and SBS21 and SBS26, respectively. An S4 (similar to SBS20) mutational signature was associated with an improved overall survival. Using maximally selected rank statistics to determine the optimal cutoff point, corresponding to the most significant relation with survival, they divided S4 into S4-high and S4-low groups, where median overall survival durations were 72 and 37 months, respectively. This finding was also validated in an independent cohort of gastric cancer patients. The main molecular and clinical features of S4-high group were predominant MSI-high status, intestinal subtype, and higher mean age, whereas, interestingly, the S4-low group was characterised by chromosomal instability (CIN) or genomically stable (GS) molecular subtype, and predominantly non-MSI status. The main inactivating mutation affecting the MMR genes in the S4 group was hypermethylation of the *MHL1* promoter. Somatic mutations of *MLH1* were found only in a few cases in S4-high, and in the S2-high and S5-high groups, which is in line with previous findings that epigenetic inactivation of *MLH1* is the main driving force in sporadic gastric cancers [22,23,61]. Their findings demonstrated that nonfunctional or absent protein MLH1 is probably the underlying cause, resulting in mutational profile of most of the cases with an S4 signature. Furthermore, the different distribution of mutated genes among the S4-high (*ARID1A*, *KMT2D*, and *TP53*) and S4-low (predominantly *TP53*) groups, and uneven distribution of MSI-high cases among these two groups indicated that different underlying mechanisms could contribute to this mutational signature, which could be of importance for prognosis. The S4-high group was also characterised by an increased number of indels in comparison with the S4-low group. It was suggested previously that the number of indels could be a good predictive biomarker for immunotherapy [108]. Additional information obtained by studying immune cell distribution and immune gene expression provided further insight in tumour surroundings in the S4-high and S4-low groups. The S4-high cases were significantly enriched with specific subtypes of immune cells, particularly those associated with cytotoxic and proinflammatory responses. Importantly, the S4-high group was characterised by a high expression of druggable targets such as *PD-L1* and *CTLA4* genes [61,63].

Immunotherapy, targeting immune checkpoints, has been approved for MMR-deficient and MSI gastric tumours; however, conflicting reports regarding the effectiveness of this therapy have been observed, ranging from only 10–20% or up to 60% of gastric cancer patients responding favourably to ICIs [65,66,67]. In addition, a subset of patients showed worse prognosis after treatment [66,67]. Research efforts, focused on finding additional, more precise biomarkers that would better predict response to ICIs in addition to MSI/PD-1/PD-L1 status, have culminated in several new findings, such as the analysis of mutational burden in plasma-circulating tumour DNA (ctDNA), immune prognostic signatures, evaluation of the composition and ratios of immune cell subtypes, and so on [16,65,71,109,110,111]. Analyses of mutational signatures associated with deficient MMR and/or MSI could provide a deeper level in understanding the mechanisms of heterogeneity found among MSI-positive gastric cancers. 

##### *POLD1*/*POLE* Mutations and MSI Phenotype 

The SBS20 mutational signature, presented in COSMIC, was correlated with concurrent *POLD1* (DNA polymerase delta 1) mutations and defective DNA mismatch repair [36]. Interestingly, this is in contrast with the S4 signature, which was described previously and is similar to SBS20; however, mutations in *POLD1* were not characteristic for S4 [61]. SBS20 was associated with ID1 and ID2 and often found in the same samples as other microsatellite instability (MSI)-associated signatures SBS6, SBS14, SBS15, SBS21, SBS26, and SBS44 [36]. SBS20 was found using WES and WGS in 11.5% (56/486) samples of patients with gastric cancer [31]. POLD1 is a catalytic subunit of the DNA polymerase delta and possesses both 3′-5′ exonuclease activity and polymerase activity. It has a crucial role in high-fidelity genome replication, acting as major processive polymerase in lagging strand synthesis and probably minor polymerase in leading strand synthesis, assuming this role particularly during replication fork stress, and in DNA resynthesis during DNA repair mechanisms, such as base excision repair, nucleotide excision repair, and homologous recombination repair [30,112,113,114]. Its damaging mutations affect genome integrity and stability and lead to accumulation of alterations in DNA, and tumour formation. The information on *POLD1*-associated mutational signature (SBS20) could therefore, in addition to other biomarkers such as MSI-high, indel-high, T-cell inflamed score-high, and PD-L1 positivity, prove valuable for identifying gastric cancer responders to ICIs [14,115,116,117,118].

A recent study that analysed mutations in *POLD* and *POLE* from the cancer patient cohort in cBioPortal observed high levels of *POLE/POLD1* mutations in several cancer types, including in 185 out of 2586 (7.2%) esophagogastric cancer samples [68]. They showed that cancer patients with *POLE*/*POLD1* mutations showed significantly longer overall survival in comparison to the wild-type population (34 vs. 18 months, *p* = 0.04), and that in addition to cancer type and MSI status, *POLE*/*POLD1* mutations were an independent risk factor for identification of patients who would benefit from ICI treatment. Similar results were observed in other studies for endometrial cancer, nonsmall cell lung cancer, and colorectal cancer, which also showed that patients with *POLD1* and *POLE* mutations might benefit from immunotherapy, more specifically ICIs, including antibodies targeting PD-1, PD-L1, or CTLA4 [119,120,121]. It should be noted that, interestingly, the number of cases with *POLE* mutations in cohorts studied in COSMIC and those in study by Buttura et al., which included 486 and 787 gastric cases, respectively, was low [36,61]. Therefore, further studies are needed to thoroughly evaluate the *POLE* mutational status in gastric cancer patients. 

##### Mutational Status of *ARID1A* and MSI Phenotype

The most unfavourable outcome in patients treated with ICIs is rapid tumour growth (hyperprogressive disease, HPD), which can occur in 10% of gastric cancer patients [67]. A recent large study indicated that *ARID1A* mutational status could be predictive biomarker for indication of favourable response to 5-FU chemotherapy combined with PD-1 inhibitors in patients with high tumour mutational burden and MSI status [70]. ARID1A is a tumour suppressor, involved in transcription by remodelling chromatin in an ATP-dependent manner and was mutated in approximately 25% gastric cancer patients. Nonfunctional ARID1A was associated with the deficiency in DNA damage response, base excision repair (BER), nucleotide excision repair (NER), MMR, HRR, overexpression of cell cycle genes and PD-L1 pathway genes, *POLE* mutations, and overrepresentation of immune cell subtypes in the tumour microenvironment [70]. Most substitutions in *ARID1A* are C > T, which are also characteristic for MMR-deficient mutational signatures SBS6, SBS15, SBS20, and SBS44, together with HRR-associated SBS3 and BER-associated SBS30 [36]. Furthermore, analysis of immune-signature revealed that tumour environments of ARID1A-deficient tumours were infiltrated with specific subtypes of immune cells, such as subsets of CD4+, CD8+ T cells and NK cells, type 17 T-helper cells (Th17), and so on. Abundant Th17 infiltration was positively associated with better overall survival and chemosensitivity [70]. The authors also established that treatment with PD-L1 inhibitors could upregulate the Th17 population in tumours, which could serve as a priming therapy for establishing chemotherapy susceptibility. This strategy, if proved to be effective in further studies, could be beneficial for MSI-inoperable tumours. In addition, since AIRD1A-mutated tumours exhibited high MSI and tumour mutation burden, the authors speculated that targeted therapy against components of DNA damage response, such as ATR or PARP, could be used in line with ICIs. It should be noted that other studies have also investigated the immune cell subsets, and particularly in the context of CD4+ and CD8+ T cells and PD-L1 expression status, there were conflicting results [71,72].

Research and clinical trials have shown that there is an unmet need for additional reliable biomarker(s) for the selection of gastric cancer patients who would benefit from ICIs. Currently, three FDA-approved biomarkers, indicative for ICIs treatment in cancers, are PD-L1 positivity by immunohistochemistry, MSI status, and tumour mutational burden, although the efficacy of the latter two has been challenged in several studies, as mentioned above. 

#### 3.3.3. Double Strand Break Repair by Nonhomologous End Joining

ID6 and ID8 were associated with defects in NHEJ, a mechanism responsible for the repair of double-strand breaks (DSBs) [31]. NHEJ directly joins two broken DNA strands by a template-independent mechanism and is active throughout the cell cycle, whereas HRR, which also repairs DSBs through a homology-dependent mechanism, is only active during the S and G2 phases [122]. DSBs are common in physiological cellular processes such as meiosis, class switch recombination, and V(D)J recombination [123]. Exogenous damaging factors, such as ionising radiation, ROS, and certain chemical compounds are also a source of DSBs. Misrepaired DSBs lead to chromosomal translocations and aberrations causing CIN, which may result in oncogenic transformation or cell death [124]. CIN is a hallmark in 49.8% gastric cancer cases, according to the TCGA study [22]. In addition to NHEJ, several other factors, such as impaired chromosome cohesion, spindle assembly, kinetochore–microtubule attachment and cell-cycle regulation contribute to CIN [125]. Nevertheless, defects and overexpression of key NHEJ proteins such as KUs, DNA-PKcs, DNA ligase IV, and XRCC4 have been reported in many cancers, including gastric cancer [76]. More importantly, defective, hyperactivated, or underactivated DNA repair could significantly affect treatment response, particularly resistance to therapy and survival outcomes, as NHEJ has a central role in radio- and chemotherapy resistance through hyperactivation of the involved proteins. Patients who showed therapy resistance or relapse and displayed overactivated NHEJ might benefit from NHEJ inhibitors. Several such inhibitors are being studied and are reviewed in a publication by Sishc and Davis [126]. For example, wortmannin, a DNA-PKcs and PI3K inhibitor, has radio-sensitising effects and was also shown to intensify the ionisation radiation effect in cancer cells [73]. LY294002, a quercetin derivative, has similar properties; however, in some studies it showed significant off-target activity [74]. NU7026 appears to be a promising compound due to its selectivity against DNA-PKcs and potency and the ability to enhance the effect of IR and etoposide [75]. Several DNA ligase IV inhibitors have also been studied, with SCR7 being the most potent one [73]. The clinical efficacy of NHEJ inhibition is under debate as this repair pathway prevents the genomic instability in normal cells through the repair of DSBs; however, NHEJ also drives carcinogenesis in cancerous or perhaps precancerous cells due to mutation accumulation in key protein members or due to the impairment of other DSBs repair pathways. Therefore, targeted cell delivery of NHEJ inhibitors should be considered in the future.

### 3.4. Reactive Oxygen Species

SBS18 is associated with damage caused by ROS [31,77]. Exo- or endogenously induced ROS generate nucleotide base damage which, if not repaired properly, can result in mutation. These include pollutants, radiation, smoking, drugs, xenobiotics, and food components. In relation to gastric cancer, a considerate amount of ROS is formed during *H. pylori* infection, mainly by neutrophils to kill the bacteria [127]. Endogenous sources of ROS are metabolic pathways in cellular organelles with high oxygen consumption, such as mitochondria, peroxisomes, and endoplasmic reticulum. 

One of the most common ROS-induced base modifications, 8-Oxoguanine (8-oxoG), can mispair with adenine during DNA replication, causing G:C > T:A transversion mutations. It has been estimated that approximately 2400 of 8-oxoG sites per cell can be found in cells without additional exposure to exogeneous carcinogens [128]. OGG1 and MUTYH enzymes are DNA glycosylases that remove 8-oxoG from 8-oxoG:C pairs and the mispaired adenine from the daughter strand, respectively [129]. Germline biallelic *MUTYH* mutations can result in *MUTYH*-associated colorectal polyposis and predisposition to colorectal cancer [130]. Carriers of bi- and monoallelic *MUTYH* mutations are also at higher risk for the development of gastric polyps and gastric cancer [77,81,82,83]. WES of *MUTYH*-associated polyposis in colorectal cancer revealed a distinct mutational pattern, SBS36 with frequent 8-oxoG:A mismatches in cancer driver genes (*APC*, *KRAS*, *PIK3CA*, *FAT4*, *TP53*, *FAT1*, *AMER1*, *KDM6A*, *SMAD4*, *SMAD2*) [131]. Although signature SBS36 was not identified in gastric cancer samples in a study published by Alexander et al. in 2020, signature SBS18, present in gastric cancer samples, has a similar profile (Pearson correlation coefficient of 0.77) and possibly indicates a similar underlying mechanism, *MUTYH* mutations and defective base excision repair, which needs further validation [31,131]. In nonsporadic colorectal cancer, defective *MUTYH* results in a relatively modest mutator phenotype; nevertheless, it is an important risk factor for colorectal cancer [132].

Mutations in *MUTYH*-associated polyposis may result in excessive neoepitopes, which are able to trigger an immune response. One such case report has been published, investigating a colorectal cancer patient, who carried two inactive *MUTYH* alleles and did not respond well to chemotherapy. However, this patient responded to the administration of PD-1 inhibitor (nivolumab), which resulted in the reduction in tumour size and metabolic activity [78]. Mouw et al. also suggested that tumours, driven by *MUTYH* mutations, might be responsive to PD-1/PD-L1 inhibitors [79]. MUTYH also plays a role in the activation and phosphorylation of CHEK1 [133]. This tumour suppressor is involved in the homologous recombination repair pathway [134]. Deleterious mutations in *CHEK1* have been associated with responsiveness to PARP-inhibitor olaparib and longer progression-free survival, together with overall survival and a longer period free from pain progression [80]. Therefore, patients with sporadic gastric cancer with SBS18 could be further evaluated for *MUTYH* mutational status or base excision and homologous repair deficiency to assess responsiveness to targeted therapies with inhibitors. It could also be worth considering including SBS18 status in surveillance programs for high-risk patients (who carry *MUTYH* mutations). It is noteworthy to mention that there is an increased risk in the development of stomach polyps and stomach cancer in individuals with hereditary MUTYH-associated polyposis [135], who predominantly develop colorectal polyps and colorectal cancer; therefore, SBS18 signature of stomach epithelia could indicate malignant changes in stomach. 

### 3.5. Helicobacter Pylori Infection

Individuals infected with *H. pylori* have significantly increased risk for gastric cancer in comparison with noninfected individuals [136]. *H. pylori* causes chronic gastric epithelial inflammation, which leads to tissue remodelling and neoplasm formation. CagA and VacA bacterial cytotoxins trigger the production of inflammatory cytokines in cells [137]. Sustained expression of CagA in gastric epithelial cells resulted in SBS3 and ID6 mutational signatures, which have been previously associated with BRCAness [84]. Interestingly, they were found in intestinal gastric cancer samples, despite the lack of *BRCA1/2* mutations [31,53]. These results suggested that CagA provokes transient BRCAness in host cells, thus causing genome instability [138]. So even after eradication of *H. pylori* in a patient, malignant transformation continues [139]. PARP inhibitors are not an option for the treatment of these tumours characterised by BRCAness, since the activity of BRCA1 is fully restored [140,141]. Additionally, whole exome sequencing of gastric cancer tissues from five individuals infected with *H. pylori* revealed enrichment in C:G > T:A transition variants, which were notably more prevalent in MSI tumours [142]. Analysis of the sequence context (GpCpNp; N any base) revealed that AID deaminase activity could presumably be involved in this mutational pattern. Additionally, this signature was also present in adjacent infected normal tissue. AID activity is strongly correlated with gastrointestinal chronic infections and tumourigenesis [85]. More experiments on infected gastric cell lines and animal models are necessary to study mutational signatures related to *H. pylori* infection. Perhaps such mutational signatures would prove useful as an early onset signature for screening and surveillance of patients at high risk for gastric cancer. 

### 3.6. Mutational Signatures of Unknown Origin

The underlying mechanisms causing around half of the catalogued mutational signatures remain unknown. In gastric cancer, these are currently the following: SBS5, SBS40, SBS17a and SBS17b, SBS28, DBS4, ID5, and ID14 [31]. SBS5, SBS40, and ID5 appear to be more or less present in all cancer types. SBS5 has previously been attributed to a continuous mutational process in normal tissues, similar to SBS1, which has been ascribed to aging [40]. SBS5 origin is not well understood, but it was proposed to be associated with continuous exposure to ubiquitous metabolic mutagen as it was more prominent in kidney cancers originating from metabolite-absorbing kidney proximal tubular epithelium in comparison to those originating from cells of the cortical-collecting duct [40,143]. However, it was also identified in cell clones when analysing mutational signatures in cancer cell lines, and it continued to exist in daughter cells, even when no exogenous mutagen was present, implying that an unknown endogenous mechanism could be responsible for this phenomenon [47]. Notably, it is increased in bladder cancer samples with mutations in *ERCC2*, a base excision repair protein. Several *ERCC2* variants have been correlated to gastric cancer risk, and *ERCC2* expression levels may serve as a marker for chemoresistance in colorectal cancer [144,145,146]. Nevertheless, there have been implications that SBS5 may be contaminated with SBS16 with unknown origin [36], so further refinements are necessary to confirm the exact aetiology of SBS5 and to evaluate its potential relation to ERCC2 in gastric cancer. DBS4 and ID5 (and SBS40 in certain cancer types) also display a clocklike feature and are correlated to age at cancer diagnosis, implying they may be endogenously generated signatures.

Significant correlation was reported between the rate of somatic retrotransposition and SBS17a/b, suggesting a potential association between these signatures and APOBEC activity [47]. Interestingly, these signatures were present in stock cell lines but did not continue to be acquired in vitro even though the parent cells were overwhelmed with these signatures, arguing that a certain exogenous factor could be responsible for the underlying mechanism. SBS28 appears to be found in a limited group of cancer types and is associated with SBS10a and SBS10b, which are likely related to polymerase epsilon exonuclease domain mutations (*POLE*). Interestingly, SBS28 contributes to very high numbers of mutations when found in samples with SBS10a/b, even though the mutation numbers are much lower in samples lacking SBS10a/b [36]. It is worth mentioning that ID14, in addition to gastric cancer, was found only in colorectal and oesophagus cancer, which may point to a molecular mechanism common to all three types of cancer [31]. This signature characteristically generates large numbers of indels with no apparent evidence of defective MMR [36]. 

## 4. Conclusions

Gastric cancer is one of the deadliest malignancies worldwide with very complex and heterogenic features. This is reflected in the fact that there has been no major progress concerning gastric cancer therapy in the past decade, leaving the majority of patients with very poor prognosis. A comprehensive insight into the genomic landscape of tumour cells would provide a better understanding of tumour aetiology and facilitate an individualised approach to patient management. 

Certain mutational signatures have already been associated with clinical outcomes and can serve as potential biomarkers for target therapies or a combination of therapies. Nonetheless, the field of mutational signatures is still in its infancy, and it is critical to avoid misclassification, misattribution, and overlapping features when investigating mutational signatures. As more complex signatures will possibly emerge from more thorough investigations, researchers should also be aware of cross-contamination due to tumour heterogenicity and between tumour and adjacent nontumour cells. It is also necessary to be mindful of the fact that biopsies may not reflect a representative sample. Experiments on cell lines and animal models together with cell and patient-derived xenograft models and organoids are necessary to fully understand the association between specific mutational signatures or a combination of mutational signatures and gastric tumourigenesis and their impact on the response to different treatments. It would also be interesting to perform studies of mutational signatures profiles linked with other important contributors to gastric tumourigenesis, such as *H. pylori* infection and hypergastrinemia. Furthermore, large independent cohorts with multiple samples from the same patient and elaborate molecular and clinical data are essential to study the associations between the tumour mutational signature profile and other biomarkers already used in clinics, along with clinical outcomes. WES- and WGS-specific signatures should also be characterised, because, although WGS gives a more comprehensive image of the mutational landscape, WES is more likely to be used in a clinical setting. It is important to understand signature penetrance and integrate genomic, epigenomic, transcriptomic, and molecular markers for a more accurate cancer subtyping with defined hallmarks and with predictive diagnostic and prognostic value in order to develop necessary frameworks to design successful patient-tailored treatment strategies. Understanding the genomic background of gastric cancer through investigating mutational signatures together with the identification of underlying molecular mechanisms and evaluation of clinical data could, therefore, aid in patient stratification and selection of the most appropriate targeted therapy.

## Figures and Tables

**Table 1:** Summary of mutational signatures in gastric cancer and their potential treatment implications.

Underlying Mechanism	Signature Type: Base Substitution Subtype ^1^ [References]	Molecular Consequences	Treatment Implications [References]	Additional Biomarkers [References]
Aging	SBS1: C > T (N**C**G) [29,31,39,40]	Deamination of 5-methylcytosine	**immune checkpoint inhibition** **(PD-L1 and PD-1 inhibitors)** [16,41,42]	senescence score, **EBV^+^**, **TMB** [43]
APOBEC overactivity	SBS2: C > T (T**C**N) SBS13: C > A (T**C**A), C > G (T**C**A, T**C**C, T**C**T) [29,31,44,45,46,47]	High mutational load in transcriptionally active genes	APOBEC3B inhibitors, immune checkpoint inhibition (PD-L1 and PD-1 inhibitors), lethal mutagenesis treatment [48,49,50,51]	**EBV^+^**, **TMB**, L1-sequencing, APOBEC3B expression levels [31,45,50,52]
Homologous recombination repair (*BRCA1/2* mutations)	SBS3: C > A, C > G, C > T, T > A, T > C, T > G (N**N**N) ^3^ ID6: microhomology—deletion length: 5+ (microhomology length: 1, 2, 3, 4, 5+) [27,29,53]	Higher mutational burden due to alternative error-prone DSBs repair by NHEJ	**Pt-based therapy**, **PARP inhibitors** [54,55,56,57]	**BRCA1/2 expression levels,** *ATM* loss [58,59,60]
Mismatch repair	SBS20 (*POLD1* mutations): C > A (C**C**C, C**C**T), C > T (A**C**A, G**C**A, G**C**C) SBS6, SBS14 (*POLE* mutations), SBS15, SBS21, SBS26, SBS44 ^2^ DBS7: AC > NN (CA), CT > NN (TC), GC > NN (AT), TA > NN (AT), TT > NN (AA, AG, CA, GA) DBS10: CG > NN (TA), TT > NN (GG) ID1: 1 bp insertion (T, homopolymer length: 5+) ID2: 1 bp deletion (C, homopolymer length: 5, 6+) [31,61]	Microsatellite instability, middle to high tumour mutational burden	**Immune checkpoint inhibition** **(PD-L1,** **PD-1 and CTLA4 inhibitors)** [62,63,64,65,66,67]	**MMR mutations**, **MSI status**, **PD-L1 status**, **EBV^+^**, **T-cell inflamed score**, **TMB status,** *POLD1* mutations, *ARID1A* mutations [22,68,69,70,71,72]
Nonhomologous end joining repair	ID6: microhomology—deletion length: 5+ (microhomology length: 1, 2, 3, 4, 5+) ID8: > 1 bp deletion at repeats—deletion length: 5+ (number of repeat units: 1); microhomology—deletion length: 5+ (microhomology length: 1, 2, 3) [31]	Chromosomal instability	NHEJ inhibitors [73,74,75]	CIN, KUs, DNA-PKcs, DNA ligase IV, XRCC4 expression levels [22,76]
Reactive oxygen species DNA damage	SBS18: C > A (A**C**A, C**C**A, G**C**A, G**C**T, T**C**A, T**C**C, T**C**T) [31,77]	DNA damage	**Immune checkpoint inhibition therapy** **(PD-L1 and PD-1 inhibitors)** **PARP inhibitors** [78,79,80]	MUTYH mutations and expression levels, *CHEK1* mutations [77,81,82,83]
*H. pylori* infection	SBS3: C > A, C > G, C > T, T > A, T > C, T > G (N**N**N) ^3^ ID6: microhomology—deletion length: 5+ (microhomology length: 1, 2, 3, 4, 5+) [31,53,84]	Inflammation, DNA damage	***H. pylori* eradication**	AID expression levels [85]

^1^ Only base substitutions with more than 5% of percentage of single base substitutions or indels (ID) with more than 5% of percentage or DBS with more than 5% of doublet base substitutions. ^2^ These SBS mutational signatures are presented in Table 2. ^3^ All substitutions in all contexts are below 2.5% of percentage of single base substitutions. Treatment options and biomarkers already implemented in clinical use for cancer treatment and diagnosis/prognosis are in bold. CIN, chromosomal instability; EBV, Epstein–Barr virus; L1, retrotransposon element; MMR, mismatch repair; MSI, microsatellite instability; N, any base; TMB, tumour mutational burden.

## Abbreviations

ACRG	Asian Cancer Research Group
BER	Base Excision Repair
CEA	Carcinoembryonic Antigen
CN	Copy Number Variations Signatures
ctDNA	Circulating Tumour DNA
DBS	Doublet Base Substitution
DSBs	Double-Strand Breaks
EBV	Epstein–Barr Virus
FDA	Food and Drug Administration
GS	Genomically Stable
HBV	Hepatitis B Virus
HCV	Hepatitis C Virus
HRR	Homologous Recombination Repair
HTLV-1	Human T-Lymphotropic Virus Type 1
ICIs	Immune Checkpoint Inhibitors
ID	Insertion and Deletion Mutational Signatures
MMR	Mismatch Repair
MSI	Microsatellite Instability
MSS	Microsatellite Stable
NER	Nucleotide Excision Repair
NHEJ	Nonhomologous End Joining
NMF	Nonnegative Matrix Factorisation
ROS	Reactive Oxygen Species
SBS	Single Base Substitutions
TCGA	The Cancer Genome Atlas
TMB	Tumour Mutational Burden
TNM	Tumour Node Metastasis
WES	Whole Exome Sequencing
WGS	Whole-Genome Sequencing
